# Air Pollution and Chronic Kidney Disease Risk in Oil and Gas- Situated Communities: A Systematic Review and Meta-Analysis

**DOI:** 10.3389/ijph.2022.1604522

**Published:** 2022-04-11

**Authors:** Ogochukwu Chinedum Okoye, Elaine Carnegie, Luca Mora

**Affiliations:** ^1^ Department of Internal Medicine, Delta State University, Abraka, Nigeria; ^2^ School of Health and Social Care, Edinburgh Napier University, Edinburgh, United Kingdom; ^3^ Urban Innovation, Business School, Edinburgh Napier University, Edinburgh, United Kingdom

**Keywords:** kidney disease, systematic review, air pollution, meta-analysis, petrochemical plants, hypertension, oil and natural gas

## Abstract

**Objective:** This systematic review and meta-analysis aimed at synthesising epidemiological data on the association between long-term air pollution and kidney-related outcomes in oil and natural gas (ONG) situated communities.

**Methods:** We synthesised studies using the PRISMA 2020 guideline. We searched databases including Medline, Cochrane Library, CIHANL, CAB Abstracts, Greenlife, African Journal Online, Google Scholar and Web of Science, from inception to April 2021. Heterogeneity across studies and publication bias were assessed.

**Results:** Twenty-five studies were systematically reviewed but only 14 were included in the meta-analysis and categorised based on the outcome studied. Residents of exposed communities have increased risk for Chronic Kidney Disease (CKD) (OR = 1.70, 95% CI 1.44–2.01), lower eGFR (OR = 0.55, 95% CI 0.48–0.67) and higher serum creatinine (OR = 1.39, 95% CI 1.06–1.82) compared to less exposed or unexposed populations. The risks for hypertension and kidney cancer between the two populations were not significantly different.

**Conclusion:** We report an increased risk for CKD and kidney dysfunction in populations residing near petrochemical plants, although from a limited number of studies. The scientific community needs to explore this environment and non-communicable disease relationship, particularly in vulnerable populations.

## Introduction

Oil and natural gas (ONG)-situated communities are exposed to severe and multiple forms of environmental degradation including air pollution [1–4]. The adverse health effects of air pollution are worse in low- and middle-income countries which coincidentally also have ineffective environmental health protection laws and regulations [5]. While there is a growing body of evidence that air pollution leads to non-communicable diseases such as respiratory and cardiovascular disease, research on air pollution and chronic kidney disease (CKD) have only received some attention in the last few years. Some researchers have reported that air pollution exposure increases the risk for chronic kidney disease, while few others report contradictory findings. Notably, most of these existing epidemiological studies have been conducted in the general population of developed countries [6]. Little is known about air-pollution associated kidney disease among people living near ONG operations despite the potential environmental health risks in these areas.

Public health and environmental sciences researchers have reported on the poor air quality and environmental degradation in communities near ONG industries which may increase the risk for NCD in residents. Additionally, residents of such communities in developing countries are often of low socio-economic status with limited access to health care [3, 7]; these multiple risk factors may combine to severely increase their risk for adverse health indices. A WHO Europe report titled “human health in areas with industrial contamination” was based on a review of studies on residents living near petrochemical plants [4]. The authors included 28 articles obtained mainly from an informal search of the PubMed database; 17 of these were conducted in China, Taiwan and the United States while only one study was conducted in Africa. The adverse health outcomes reported included haematopoietic malignancies, lung cancer, respiratory diseases, bladder cancer, and prenatal conditions; but none on kidney disease. Although the authors concluded that there was consistent evidence of air pollution associated with lung cancer and respiratory diseases, they pointed out that exposure assessment was generally uncertain in most studies.

A growing body of evidence from both toxicologic and epidemiological studies suggests that there exists an association between air pollution and respiratory diseases. The lungs, which are the primary receivers of air pollutants, share specific characteristics with the kidneys, such as inflammatory response and antiPLA2 receptors [8, 9]; and this should raise suspicion for possible adverse kidney effects. The inflammatory response induced by air particulate matters in the lungs contribute to kidney damage through a spill of lung inflammation into the circulatory system; furthermore, particulate matter (PM) has been shown to directly initiate inflammation in the kidneys [8, 9]. The biological plausibility of air pollution associated kidney disease and reports of poor ambient air quality near petrochemical industries justify this current systematic review of epidemiological research investigating the association of residing near ONG industries and kidney disease.

The researchers hypothesise that persons living near ONG operations are exposed to high levels of air pollutants which may be associated with increased risk for CKD. For this reason, this review focuses on summarising epidemiological research evidence on the association between long-term air pollution and kidney-related outcomes in *ONG* situated communities. Our review question is framed based on the PECO statement as follows:

**P**opulation- In humans living in Oil and Gas (ONG) situated communities.
**E**xposure- Is long-term exposure to high levels of air pollutants.
**C**omparator- compared to those who are less exposed.
**O**utcome- associated with adverse *kidney-related* health outcomes?

Kidney-related health outcomes include CKD, end-stage renal disease (ESRD), proteinuria/albuminuria, reduced renal function (based on estimated glomerular filtration rate), kidney cancer, hypertension, and diabetes.

## Methods

The study protocol was registered and published in the PROSPERO database in May 2021 (PROSPERO ID: CRD42021256716) [10].

### Search Strategy

The researchers identified studies conducted in oil and gas situated communities that provided information on the kidney-related outcomes earlier outlined. Relevant records were identified by searching electronic databases including Medline, Cochrane Library, CIHANL, CAB Abstracts, Greenlife, African Journal Online, Google Scholar, and Web of Science, from inception to April 2021. OO carried out a snowball search of the reference lists of selected journal articles and a review of citation lists. In addition, reference lists of review articles and risk assessments were screened for relevant literature.

The primary search was on Medline, CINAHL, CAB Abstracts, and Greenlife combined on the EBSCO search platform to identify relevant records using a combination of keywords, including the type of exposure, health outcome, and context. Additionally, a search was conducted on all other databases outlined earlier. The core search terms were *renal insufficiency chronic OR chronic kidney disease OR kidney failure OR end-stage renal disease AND Particles OR* “*particulate matter*” *OR* “*sulfur dioxide*” *OR* “*sulphur dioxide*” *OR* “*nitrogen oxide*” *OR* “*nitrogen dioxide*” *OR* “*carbon monoxide*” *OR* “*ozone*” OR air OR gas OR oil or petroleum AND pollut* AND africa OR subsaharan africa OR global OR low income OR middle income OR developing countries. Initial search included all search terms and subsequently broadened by removing the geographic search terms and kidney disease descriptors in two further steps. This allowed us identify all relevant papers on *air pollution near petrochemical or oil plants and any health outcome* , thereby capturing studies on hypertension, diabetes mellitus and other pre-specified kidney-related outcomes. The search strategy is attached as Supplementary File S1.

### Study Selection and Screening

All references identified during the search were imported to Endnote version X9 reference manager software, saved in one library and duplicates removed. Two authors (OO and EC) independently screened through the titles and abstracts of the papers obtained applying the pre-specified inclusion criteria. After that, the full texts of selected articles were retrieved and independently reviewed by two authors (OO and EC) for final selection; disagreements were resolved through discussions with the third author (LM). There was no need to contact specific authors since we had access to all included articles.

### Inclusion Criteria

The systematic review included observational epidemiological studies that met the following characteristics: involved human participants only, conducted in population located near ONG or petrochemical operations (upstream or downstream), assessed kidney-related outcomes (hypertension, diabetes, CKD, reduced GFR, serum creatinine, and kidney cancer) or reported prevalence, incidence or mortality rate of kidney-related outcome(s). Studies were included in the systematic review regardless of whether measurements or estimates of specific air pollutants or air quality for the population were reported. Only original journal articles (published or accepted for publication) written in English and published from the journal’s inception until April 2021 were included.

Meta-analysis: Of the studies included in the systematic review, those that provided a statistical effect size for the association between air pollution and health outcome or provided data for computing same were included in the meta-analysis.

### Exclusion Criteria

Studies in which authors measured the indirect effects of air pollution on health outcomes (e.g., income, community stressors) were excluded. Studies not including original data or observations, such as review articles, commentaries, editorials, anonymous reports, conference abstracts, were excluded.

For the meta-analysis, studies that did not provide effect estimates or data to compute same were excluded. Outcomes for which only one study was found was excluded.

### Data Extraction

The authors extracted data from selected studies using an Excel sheet, details of this have been published elsewhere [10]. The data extraction sheet was piloted using four randomly selected papers and adjusted. Data were extracted by OO and checked by EC, and all authors discussed disagreements for resolution.

### Risk of Bias Assessment

The risk of bias assessment was done using the Newcastle-Ottawa scale modified for cross-sectional studies [11] the Joanna Briggs Institute (JBI) quality appraisal checklists [12], and the National Toxicology Program Office of Health and Assessment and Translation risk of bias rating tool (NTP-OHAT) [13]. The Newcastle-Ottawa scale has a maximum of 10 points distributed in three sections: selection (5-points), comparability (2-points) and outcome (3-points). In each of these sections, some items are provided to appraise the articles. The authors slightly modified the points for ecological studies since one of the items in the “selection” section (i.e., *non-respondents*) was not applicable; therefore, the maximum point for ecological studies was reduced to nine. Summary scores awarded to individual studies were: 0–4 = unsatisfactory, 5–6 = satisfactory, 7–8 = good, and 9–10 = very good; and for ecological studies 0–3 = unsatisfactory, 4–5 = satisfactory, 6–7 = good, and 8–9 = very good. The JBI checklist has eight questions with one score each; a score of <4 was regarded as high risk (poor quality) while a score of 4 or greater was low risk (good quality). The NTP-OHAT rating tool has 11 questions or domains however only seven of these apply to observational studies and were used in our assessment. These questions assess *selection*, *confounding*, *attrition/exclusion, detection, selective reporting and other potential threats to validity such as statistics.* Answer format to each question include: definitely low risk, probably low risk, probably high risk or definitely high risk. Grading was done by OO and checked by EC and LM, details are attached in Supplementary File S2.

### Outcome Measurement

Outcomes were based on a physician’s diagnosis or defined by standard criteria; details are available in the previously published protocol [10].

### Synthesis of Results

Most effect estimates were pooled using the random-effects meta-analysis of DerSimonian and Laird [14], assuming inter-and intra-study heterogeneity. The fixed effect model was used for two similar studies [15, 16] which we assumed one true-effect size and that the differences among the studies was purely due to random error. Pooled summary estimated were presented as the risk ratio. All estimates were pooled according to the previously stated kidney-related outcomes. Measures were taken to prevent bias due to multiple inclusions of one city data. Where various cities are included in one study, we first generated a pooled effect estimate combining effect sizes from all cities studied before inclusion in the overall meta-analysis. The same process was done for studies in which pooled estimates were presented according to gender. Sub-group analysis was performed to compare the effects sizes from countries in the Global South [17] and North where there were sufficient studies. Heterogeneity of effect size was assessed using the *x* ^*2*^ test on the Cochran’s *Q* statistic and quantified by calculating the *I* ^*2*^ statistics [18]. The Egger’s regression test was used to check for publication bias of included studies. Sensitivity analysis was not performed due to the small number of studies. Data analysis was done using the Comprehensive Meta-analysis (CMA) v3 and RStudio (meta-package).

The GRADE guidelines [19] were used to summarise the overall rating of confidence in effect estimates for all outcomes considered (Supplementary File S2). Individual domains rated include *risk of bias*, *inconsistency*, *indirectedness*, *imprecision*, *and publication bias.*

### Reporting

This review is reported based on the Preferred Reporting Items for Systematic Review and Meta-Analysis statement (PRISMA) guideline [20].

## Results

### Study Selection

Twenty-five out of 1,768 screened articles were included, while others were excluded based on pre-set criteria. The main reasons for exclusion were: studies not conducted near ONG or petrochemical plants, and adverse health outcomes studied not kidney-related. Publication dates of included articles spanned from 1980 to 2021; however, 22 out of 25 studies were published after 2000. Ten studies (40%) were conducted in the Global South, eight of these being in Nigeria [21–28], one in Brazil [29], and one in Ecuador [30]. The majority of the studies (*n* = 15) were conducted in Urban settings in developed countries, including Taiwan, Italy, United States, and Spain. The predominant study designs were cross-sectional (*n* = 13) and ecological (*n* = 11), with one case-control.

**FIGURE 1:**
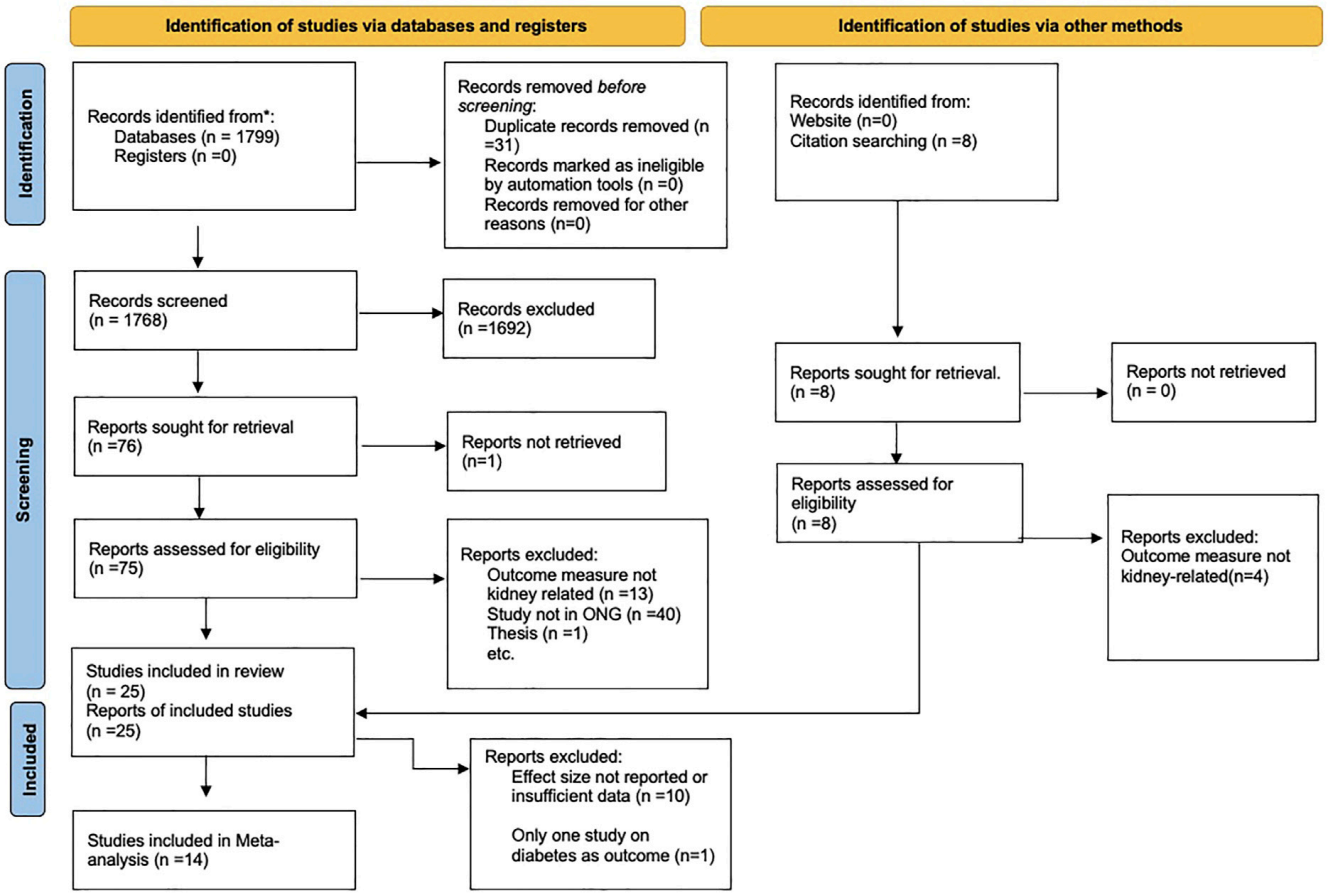
Preferred Reporting Items for Systematic Reviews and Meta-analyses (PRISMA) Flow diagram for study inclusion (Nigeria, 2021).

A summary of the inclusion process is presented in the PRISMA flow diagram for study inclusion (Figure 1), while an overview of the characteristics of all 25 studies is shown in Supplementary File S3.

### Risk of Bias Assessment

The authors appraised the quality of the 25 included studies using the Newcastle-Ottawa scale modified for cross-sectional studies [11] the Joanna Briggs Institute (JBI) quality appraisal checklists [12], and the NTP-OHAT rating tool. OO appraised the articles and categorised them based on the risk of bias, while EC and LM checked this separately, and all authors agreed. Twenty-three out of the 25 articles were classified as *low risk* using the JBI checklist. Using the Newcastle-Ottawa scale, seven studies were *unsatisfactory*, six were *satisfactory*, nine were *good*, and three were *very good.* The NTP-OHAT rated a majority of the studies as *probably* or *definitiely high-risk* in most of the domains except “selective reporting.” Supplementary File S2 shows the details of the risk of bias ratings.

### Exposure Measure

The exposure was validly estimated in only seven studies, while others assessed exposure based on the location of residence only (Supplementary File S3). Five studies estimated air pollutants using either modelling systems or existing air monitoring data, while two studies measured the urinary content of petrochemical metals including arsenic, nickel, chromium and vanadium [15, 16]. Kaldor et al. (California) and Benedetti and others (Italy) estimated air pollutants using modelling systems for air dispersion. The studies conducted in Spain, Estonia, and Serbia used the existing air monitoring database [31–33].

### Outcome Measure

Eleven studies were ecological, investigating the incidence rate or mortality rates of kidney cancer (and other cancers) in exposed versus less- or un-exposed populations. In seven other studies, the kidney outcomes measured included serum creatinine [15, 16, 21, 23, 27], serum cystatin C [27], eGFR [15, 16], the prevalence of CKD [15, 16] or symptoms of kidney disease based on ICD-9 [34]. Hypertension or blood pressure was the health outcome in eight studies, while diabetes or blood sugar was measured in three [26, 33, 35]. Only eight studies adjusted for some confounding factors such as age, sex, income per family member, education, marital status, family history of hypertension, sleep deprivation, overweight and obesity, body mass index (BMI), environmental tobacco smoke, smoking history, lifestyle factors and past occupational exposure. See details in Table 1 and Supplementary File S3.

**TABLE 1:** Characteristics and results of studies included in the meta-analysis (Nigeria, 2021).

	Author	Year	Place	Exposure	Outcomes					Adjustments for confounding Factors
					CKD	EGFR	SERUM CREATININE	HTN/Blood pressure	Kidney *Cancer* Incidence/mortality	
					OR (95%CI)	OR (95%CI)	OR (95%CI)	OR (95%CI)	HR, RR (95% CI)	
1	Abia et al	2019	Nigeria	Gas flare (not specified)			11.23 (3.24, 38.90)			
2	Alexander et al	2014	United States	Natural gas plant (not specified)					0.91(0.81–1.02); 0.97 (0.80–1.17)	age, sex, and race
3	Ejimofor et al	2016	Nigeria	Gas flare (not specified)				4.85 (1.84, 12.8)		age, education, and marital status, family history of hypertension, sleep deprivation, overweight and obesity, and lifestyle factors
4	Garcia-Perez et al	2016	Spain	VOC, PAC, PM					1.97 (1.13–3.43)	Age, sex, level of education, marital status, occupation, BMI
5	Hurtig et al	2002	Ecuador	oil exploration, not specified					2.78 (0.49, 15.78)	
6	Maduka et al	2017	Nigeria	Gas flare (not specified)				1.75 (1.11, 2.75)		Sex, age, BMI, marital status, level of education, occupation
7	Odo et al	2019	Nigeria	Gas flare (not specified)			273.81 (129.97, 576.86)			
8	Orru et al	2018	Estonia	benzene, phenol, PM				1.10 (0.98, 1.24)		Gender, age, BMI, environmental tobacco smoke (ETS), smoking history, and income per family member
9	Pirastu et al	2013	Italy						1.34 (1.19, 1.50)	Deprivation index
10	Ribeiro et al	2016	Brazil	Industrial sources (PAH, organochloride pesticides, PCB, dioxins and furans				1.22 (1.08–1.37)		age, time of residence, education, local egg consumption, local mollusks consumption, past occupational exposure
11	Salerno et al	2012	Italy	Petrochemical (not specified)					4.28 (1.16, 20.10)	
12	Yang et al	1997	Taiwan	Petrochemical (not specified)					0.86 (0.54, 1.37)	
13	Yuan et al	2021	Taiwan	Petrochemical metals-nickel, chromium and vanadium	2.70 (1.96, 3.72)	0.35 (0.26, 0.46)	1.20 (1.01, 1.42)			age, sex, BMI, education level, smoking habits, diabetes mellitus, and living near a major road
14	Yuan et al	2020	Taiwan	Petrochemical - arsenic, PAH	1.47 (1.21, 1.78)	0.65 (0.55, 0.77)	1.59 (1.20, 2.10)			age, sex, BMI, education level, smoking habits, diabetes mellitus, and living near a major road

CI, confidence interval; HR, hazard ratio; OR, odds ratio; RR, relative risk; BMI, body mass index.

### Narrative Summary of Study Characteristics and Results

Supplementary File S3 summarises the main characteristics and results of all 25 studies. Twelve studies showed that exposure to air pollution was associated with increased risk for kidney-related outcomes. Three studies reported that exposed populations had a lower risk of kidney cancer [31, 36, 37]. One study reported no significant difference in the risk for hypertension or diabetes between exposed and unexposed populations [33], while the remaining nine studies reported mixed findings.

Of the 12 positive studies, four studies found higher blood pressures or increased odds of hypertension in the exposed population compared to the less exposed. Other studies reported higher serum creatinine (*n* = 3), higher serum cystatin C (*n* = 1), reduced eGFR (*n* = 2) or higher prevalence of CKD (*n* = 2) in the exposed population compared to unexposed or less exposed. One study found higher blood sugar levels in those exposed [26]. Of these 12 studies, three provided data for exposure assessment [15, 16, 32] while others used the location of residence only; effect size was provided in 10 studies. In three studies, authors reported that persons living in exposed areas had a lower incidence of kidney cancer or mortality rates than reference populations; only one of these studies provided air monitoring data and effect sizes [31].

Findings were mixed in nine studies; six of these were on kidney cancer incidence rates or mortality rates in exposed vs. less exposed populations and findings varied based on sex and race. For instance, in the Louisiana, United States study, there was no statistically significant difference in age-adjusted mortality rates among exposed vs. unexposed non-whites, but a lower or higher mortality rate in exposed white males depending on the decade of study, the 1980s’ or 1990s’ [35]. Similarly, in the Taiwanese study, authors found that the age-adjusted mortality rate was lower in exposed males but higher in females, although not statistically significant [38]. Conversely, another study found a higher hospitalisation rate for kidney disease among exposed males and younger persons but not in females and those aged >60 years [34]. Pirastu et al. found significantly higher age-adjusted mortality and hospitalisation rates for kidney cancer in a few districts studied compared to a reference town, but this was not the case in other districts [39]. Of these nine studies with mixed results, effect sizes and exposure assessment data were not provided in six.

#### Findings Based on Country Level of Development

Ten studies were conducted in developing countries; eight showed that exposure resulted in adverse kidney outcomes. Two studies showed mixed findings [29, 30], and none showed a reduced risk of kidney disease among exposed persons. Conversely, of the 15 studies conducted in developed countries, five showed an increased risk for kidney-related outcomes in exposed persons, four studies showed a reduced risk of kidney outcomes, while six studies revealed mixed findings. Twelve out of the 15 studies conducted in developed countries reported exposure assessment, while none of the studies conducted in developing countries reported exposure assessment.

### Pooled Effect Estimates

We included only 14 out of the 25 studies (56%) in the pooled effects estimation based on prior criteria (Table 1). Other studies were excluded for the absence of effect estimates, insufficient data, and presence of only one study. Ten of the 14 studies had effect sizes, including the odds ratios (OR) and 95% confidence intervals in five studies, relative risks (RR) in four, and hazard ratios (HR) in one. Four other studies provided means and standard deviation of outcomes including serum creatinine and eGFR; the OR was computed using the CMA software.

The included studies were categorised based on outcome measures as follows: hypertension (*n* = 4), CKD (*n* = 2), renal function including eGFR (*n* = 2) and serum creatinine (*n* = 4); kidney cancer mortality (*n* = 3), kidney cancer incidence/hospitalisation (*n* = 3). Only one study presented an effect size for diabetes outcome, showed that people living in areas with the highest quartile level of exposure did not have significantly higher odds of diabetes OR = 1.01 (0.85, 1.21).

#### Hypertension

**FIGURE 2:**
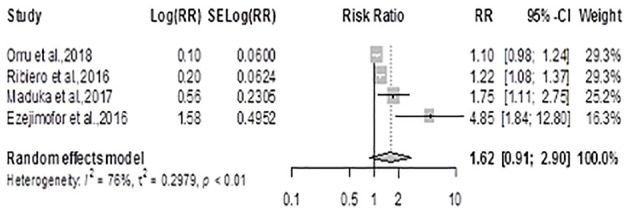
Forest plot and pooled effect estimates of the association between hypertension and high exposure to air pollutant (Nigeria, 2021).

The pooled effect estimate (ES) for hypertension using the random-effects model was 1.62 (0.90, 2.90), *p* = 0.10, showing no statistically significant difference in the risk for hypertension between exposed and unexposed residents, Figure 2. There was considerable heterogeneity across studies (Q *= 12.67*, *df = 3*, *p = 0.005*; *I* ^*2*^ *= 76.3%*)*.*

#### Chronic Kidney Disease

**FIGURE 3:**
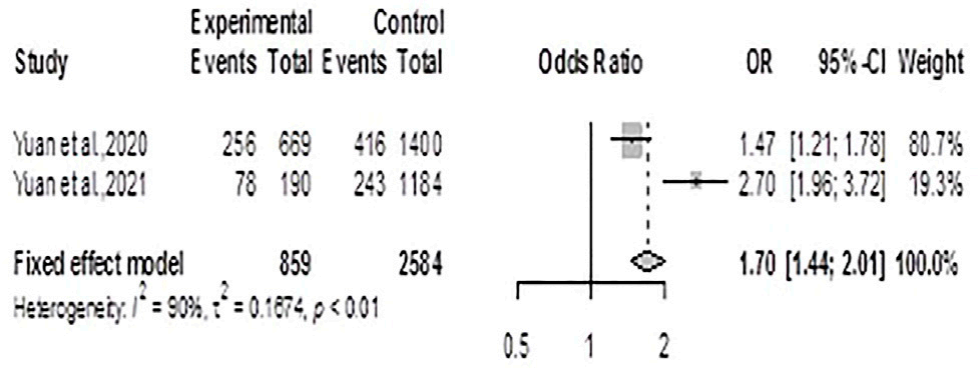
Forest plot and pooled effect estimates of the association between high exposure to air pollutant and chronic kidney disease (Nigeria, 2021).

Using the fixed effects model, the meta-analysis for CKD generated an ES = 1.70 (1.44, 2.01), *p* < 0.0001 showing that residents of exposed communities had significantly increased risk for CKD, Figure 3. There was significant heterogeneity between the two studies included (*Q =10.13*, *df = 1*, *p-value = 0.0015*; *I* ^*2*^ *= 90.1%*)

#### Renal Function

**FIGURE 4:**
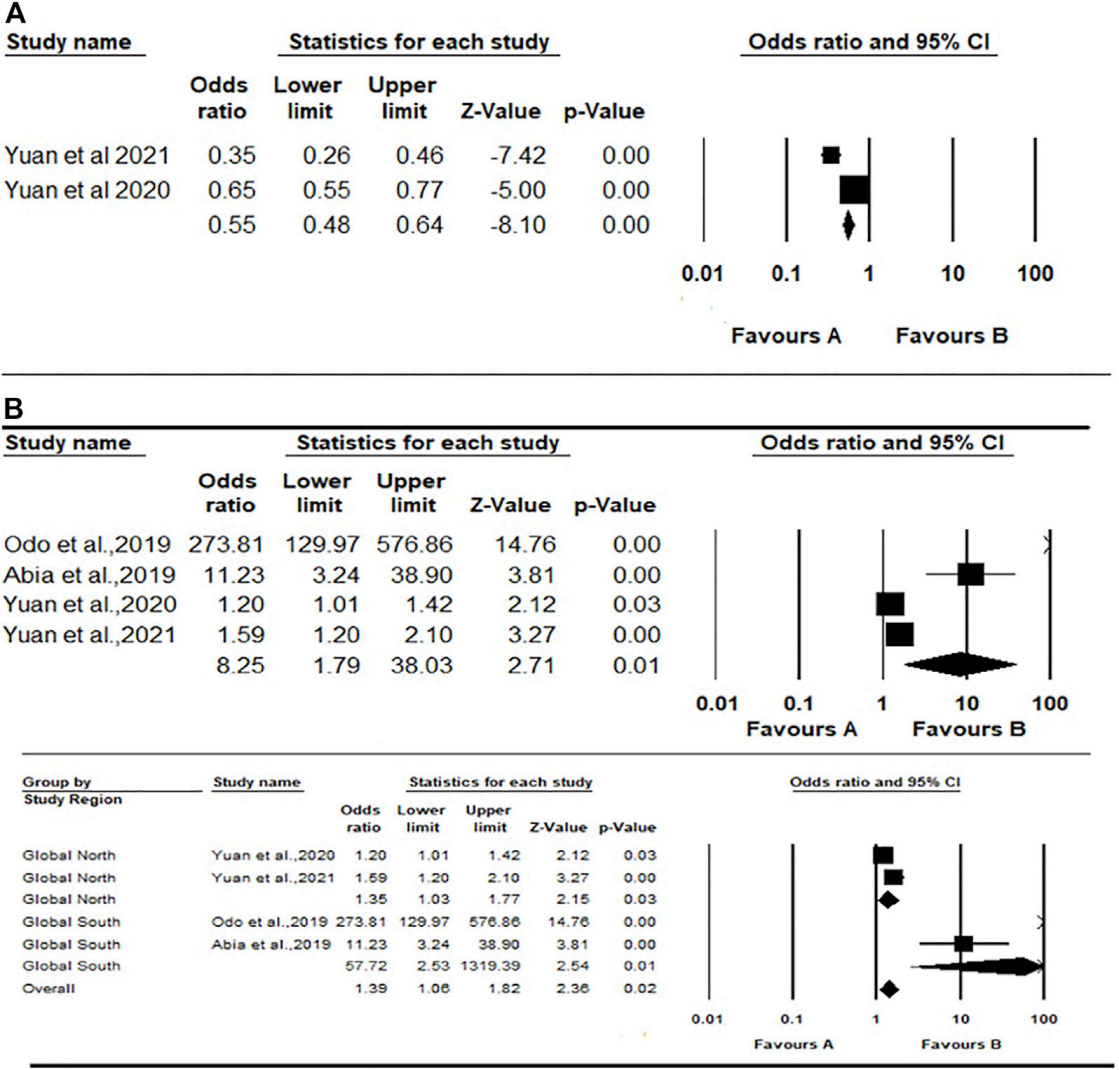
Forest plot and pooled effect estimates of the association between high exposure to air pollutant and estimated glomerular filtration rate **(A)**; and the association between high exposure to air pollutant and serum creatinine **(B)** (Nigeria, 2021).

The meta-analysis for eGFR using the fixed effects model generated ES = 0.55 (0.48; 0.64); *p* < 0.0001, meaning that residents of exposed communities have 45% reduced risk of having high eGFR compared to unexposed communities, Figure 4A. There was significant heterogeneity between the two studies included (*Q* = 14.45, *df* = 1, *p*-value < 0.0001; *I* ^*2*^ *= 93.07*).

The meta-analysis for serum creatinine using the random-effects model showed a pooled estimate of 8.25 (1.79; 38.03)**,** *p* < 0.007, meaning that residents of exposed communities were more likely to have higher serum creatinine and therefore increased risk for kidney disease (Figure 4B). There was considerable heterogeneity across the studies included (*Q = 203.87*, *df = 3, p-value < 0.0001*; *I* ^*2*^ *= 98.5%*). A further mixed-effect model was conducted due to the large effect sizes reported by the two included Global South studies (OR = 273.60 and 11.22 respectively) compared to the two studies from the Global North (OR = 1.19 and 1.59 respectively). This analysis generated an overall pooled estimate of 1.38 (1.05, 1.82), *p* value = 0.018. The pooled effect estimate for the two *Global North* studies was 1.35 (1.03–1.77), *p* = 0.032; while the summary effect for the *Global South* studies was 57.70 (2.52–1,317.92), *p* = 0.011 (Figure 4B).

#### Kidney Cancer

**FIGURE 5:**
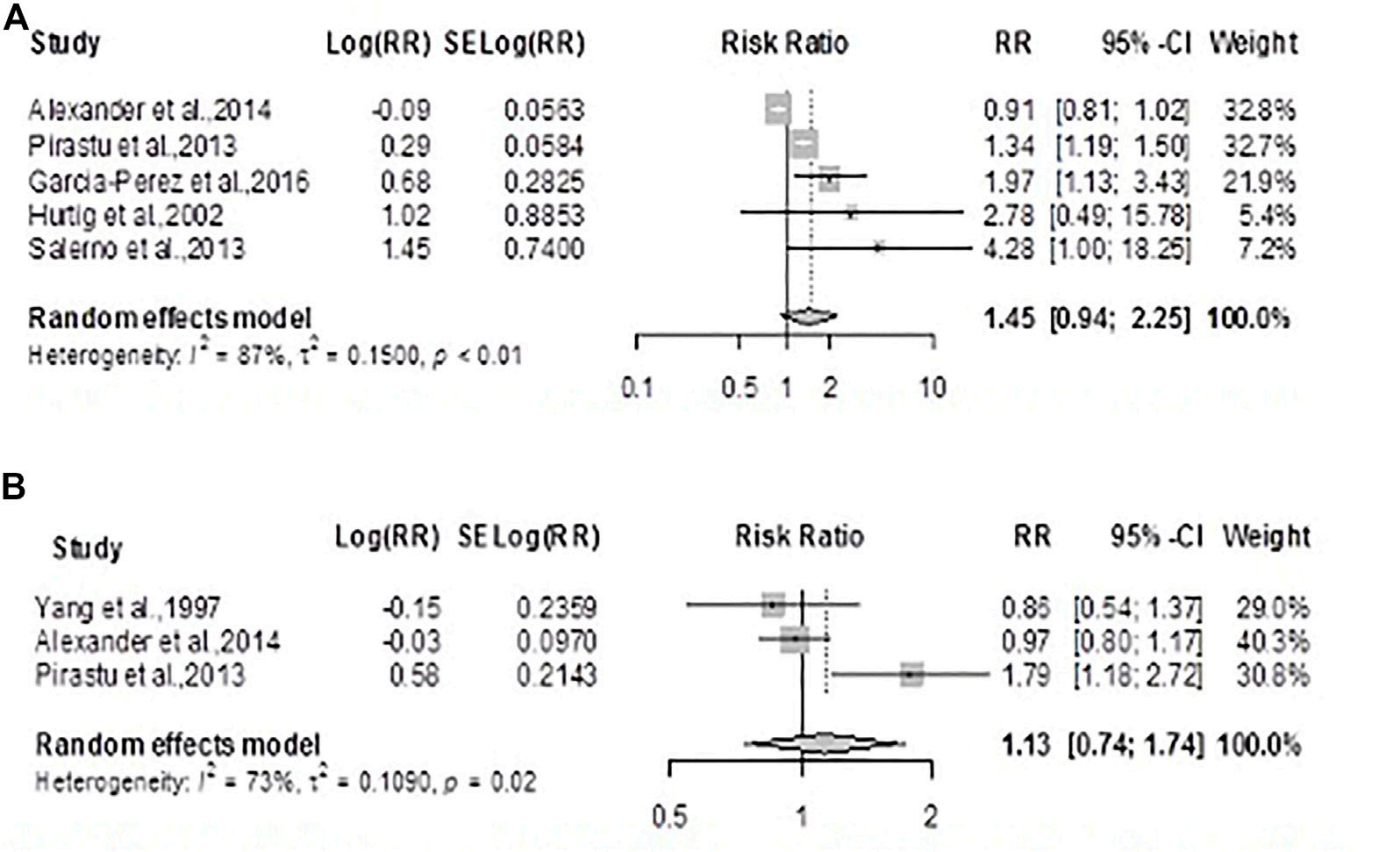
Forest plot and pooled effect estimates of the association between high exposure to air pollutant and kidney cancer mortality **(A)**; and kidney cancer incidence **(B)** (Nigeria, 2021).

The summary RR for kidney cancer mortality using the random-effects model was 1.13 (0.74; 1.74) *p* = 0.56, showing no statistically significant difference in the risk for cancer deaths between exposed and unexposed residents (Figure 5A). There was considerable heterogeneity across studies (*Q* = 7.54, *df* = 2, *p* = 0.0230; *I* ^*2*^ *= 73.5%*). The summary RR for kidney cancer incidence using the random-effects model was 1.45 (0.93, 2.25), *p* = 0.09 showing no statistically significant difference in the risk for kidney cancer between exposed and unexposed residents, Figure 5B. There was also considerable heterogeneity across studies (*Q =31.35*, *df = 4, p-*value *< 0.0001; I* ^*2*^ *= 87.2%*)*.*

### Publication Bias

Publication bias was evaluated using Egger’s regression test and did not produce statistically significant results for all the analyses performed. Noteworthy is that the small number of studies included in the test may have affected its ability to detect the probability of a small study effect.

The GRADE guideline was used to make an outcome-specific overall rating of confidence in effect estimates (Supplementary File S2). The overall ratings for all outcomes were either “low” or “very low” quality and this was mainly because all the studies were observational (cross-sectional and ecological) and so had a “low” baseline rating. Additionally, exposure assessment was not valid in most of the studies; confounding factors were not adjusted and there were *inconsistencies (heterogeneity*, *point estimates varying widely and confidence intervals showing no overlap)* in a considerable number of the studies.

## Discussion

We synthesized existing epidemiological literature on the association between air pollution in communities near ONG plants and kidney-related outcomes to determine whether residents are at greater risk for kidney disease. Our meta-analysis shows that residents of exposed communities have an increased risk for CKD, lower eGFR and higher serum creatinine compared to less exposed or unexposed populations. The chances for hypertension and kidney cancer (incidence and mortality) in exposed populations were not significantly different from unexposed or less exposed reference populations. However, studies included in this synthesis were small in number and considerably heterogeneous.

### Kidney Cancer

We found relatively more studies regarding the association between living in exposed areas and kidney cancer incidence or mortality compared to other kidney-related outcomes. Still, these were mainly ecological studies with the drawback of *ecological fallacy*. Mixed observations which varied based on gender and race suggest that the adverse effects of air pollution on health depend not only on the physical characteristics of the pollutant but also on certain host factors, which has been earlier proposed [40]. Surprisingly, a few studies favoured a reduced risk for kidney cancer in exposed populations and the reason for this is unclear.

Scientists affiliated with the WHO’s International Agency for Research on Cancer synthesized 20 studies and similarly reported no statistically significant difference in the risk for kidney cancer mortality [ES = 1.03 (0.91, 1.16)] or incidence [ES = 1.04 (0.81, 1.32)] between the exposed (petroleum industry workers) and unexposed [41]. Furthermore, the pooled estimate for two nested-case control studies on kidney cancer incidence among residents of oil-producing communities was also not statistically different [ES = 1.55 (0.84, 2.83)]. Notably, the cohort studies included in their analyses had sufficiently long follow-up period ranging from 4 to 77 years. The authors reasonably argue that petroleum workers studied may have been as healthy or healthier than reference populations because industries employ only *healthy workers* who pass pre-employment medical examinations. They noted that there was limited literature on the subject (none arising from Africa); exposure assessment was crude in most studies, and that most studies often reported on multiple cancer sites with varying frequencies, which can affect the power of analysis and risk determination.

Another systematic review on the risk for urological cancers in exposed general populations included only one study on kidney cancer incidence and mortality, respectively [42]. They similarly reported a non-significant increased risk of kidney cancer in persons exposed to NO_x_, PM_10,_ PM_2.5_, and NO_2_, however observed increased mortality among persons exposed to PM_2.5_ [HR = 1.14 (1.03, 1.27)] but not O_3_ [HR = 0.97 (0.86, 1.09)]. This review, unlike ours, included only studies that reported incremental measurement of air pollutants and effect estimates; this may possibly explain the lack of studies.

#### Chronic Kidney Disease and Reduced Kidney Function

Only four studies included in our meta-analysis explicitly focused on kidney function (serum creatinine, eGFR) or CKD outcomes [15, 16, 21, 27]; and all reported an increased risk for adverse kidney outcomes in exposed populations. These findings suggest that exposure to air pollution is a cause of kidney damage; however, the certainty of data from these studies ranged from weak to vigorous. These studies were all cross-sectional and did not include measurements of air pollutants, although Yuan et al. measured the urinary content of some petrochemical metals. Furthermore, only two studies adjusted for confounders so these results should be interpreted with caution.

A previous meta-analysis of observational studies on the association between long term exposure to gaseous or particulate matter and incident CKD, ESRD or renal dysfunction in general populations reported an increased risk for CKD in persons exposed to PM_2.5_ [1.10 (1.00–1.21)], PM_10_ [1.16 (1.05, 1.29)] and NO_2_ [1.11 (1.09, 1.14)] [6]. Furthermore, 10 µg/m^3^ increases in PM_10_ and PM_2.5_ were associated with eGFR decline by −0.83 and −4.11 ml/min, respectively. This meta-analysis included only 14 studies, although only 2–4 studies were included per air pollutant reported. Unlike most other studies cited, the researchers excluded studies that did not adjust for confounding variables; however, they acknowledge the possibility of residual confounders or effects of unmeasured confounders. Other authors have reported similar reported increased risk of CKD associated with PM and NO_2_ [43].

#### Hypertension

The pooled estimate from this study showed no statistically significant difference in the risk for hypertension between populations residing near petrochemical industries and unexposed reference populations. However, three out of the four studies individually reported a significantly increased risk for hypertension in exposed populations. The varying results from individual studies are possibly due to factors related to the population studied and the study methods; studies with relatively higher weighting reported smaller effect sizes with narrower confidence intervals than others, which determined our overall pooled estimates. A previously published meta-analysis of 100 studies based on general populations reported an increased risk for hypertension associated with long-term exposure to PM_2.5_ [OR = 1.05 (1.01, 1.09)], and higher DBP associated with long-term exposure to PM_10_, PM_2.5_, and NO_2_ (ß values: 0.47–0.86 mmHg) [44]. The observed increased risk for high blood pressure/hypertension in exposed populations further strengthens the argument for increased CKD burden in these populations, hypertension being a leading cause of CKD. The large number of studies in this review confirms that while there is ample data on air pollution and cardiovascular outcomes, few studies exist on kidney outcomes.

It is noteworthy, that studies conducted in developing countries all reported a positive association between exposure and kidney-related outcomes but this was not so with the studies from developed countries. This observation suggests that the effect of air pollution is worse in the less-endowed population, a finding that has been previously offered [45]. However, it is vital to consider the certainty of these studies; none of the studies from developing countries measured exposure, and the majority did not adjust for confounders.

Finally, most of the studies included in our review did not carry out an exposure assessment but instead relied on the location of residence. None of the studies measured air pollutants in real-time; some studies estimated exposure using modelling methods or land use regression but did not provide incremental measures of air pollutants during statistical analysis. Although it is common practice among epidemiological researchers to assess environmental exposure by location, this has certain drawbacks and has been frowned at by researchers. This crude method’s validity, accuracy, and utility have been questioned; concerns raised include issues relating to “multiple exposure pathways, persistent and toxic contaminants, and cumulative exposures from non-point, mobile and point sources” [46]. There should be no ambiguity regarding exposure assessment and outcome measurement when the aim is to establish causality.

### Conclusion and Recommendations

This systematic review and meta-analysis showed an increased risk for CKD and kidney dysfunction in populations residing near petrochemical plants. Our findings should serve as a *prompt to action* for all stakeholders. There is a need for more effective government policies and implementation of the same. At the individual level, there is a need to embrace health consciousness and healthy behaviours while avoiding unhealthy social habits and other practices that may be additional sources of outdoor and indoor air pollution. The scientific community needs to explore this environment and non-communicable disease relationship further, particularly in vulnerable populations. Although more efforts have been made in the last decade, the available evidence is still insufficient to establish a causal link between air pollution and kidney disease; there is a need for improved study methods and by extension, certainty of data.

### Strengths and Limitations

This systematic review and meta-analysis is the first to our knowledge to report on the association of ambient air pollution and kidney-related outcomes in residential areas near ONG industries. The main limitations of this review are the small number of included studies and the heterogeneity of studies. Most studies did not measure exposure validly, and where this was done, data on incremental measurement of specific air pollutants was not included in the analysis; these are potential sources of bias. Several studies did not adjust for confounders; therefore, our findings should be interpreted cautiously. Furthermore, it was impossible to conduct sensitivity analysis and sufficient sub-group analysis due to the limited number of studies, although included studies showed no publication bias.

This review is unique because it included studies irrespective of whether air pollutant(s) measurement was done; for this reason, we could present a valuable narrative of methods and results from studies not selected for meta-analysis. We also selected studies regardless of whether an effect estimate was reported; this allowed us to compute effect sizes for studies that provided sufficient information. We assessed the risk of bias using three standard instruments, thereby providing some insight into their strengths and limitations. Despite its limitations, our study is probably the first that included studies from across the globe with a fair representation of the Global South; this factor is often lacking in existing reviews.

## Abbreviations

CI, Confidence interval; CKD, Chronic kidney disease; CMA, Comprehensive meta-analysis; CO, Carbon monoxide; eGFR, Estimated glomerular filtration rate; ESRD, End-stage renal disease; ES, Effect estimate; GFR, Glomerular filtration rate; HR, Hazard ratio; NOX, Nitric oxide and nitrogen dioxide; NO2, Nitrogen dioxide; O_3_, Ozone; ONG, Oil and natural gas; OR, Odds ratio; PM, Particulate matter; RR, Risk ratio or Relative risk; SO2, Sulphur dioxide; WHO, World health organisation.
