# Supplementary material for: Air Pollution and Chronic Kidney Disease Risk in Oil and Gas- Situated Communities: A Systematic Review and Meta-Analysis
Source: Int J Public Health. 2022 Apr 11;67:1604522. doi: 10.3389/ijph.2022.1604522 (PMC9035494; doi:10.3389/ijph.2022.1604522)
Supplement: Supplementary file 1 [file DataSheet3.docx]

**Table S1: Characteristics of included studies**

| **S/N** | **Author** | **Year** | **Location** | **Design** | **Duration (yr)** | **Pollutant** | **Outcome measure** | **Exposed population** | **Exposure measure** | **Result and Effect Size** |
| --- | --- | --- | --- | --- | --- | --- | --- | --- | --- | --- |
| 1 | Abia et al | 2019 | Nigeria | Cross-sectional | 0 | Gas flare, not specified | serum creatinine | rural Low SES M=20, Reference town: Rural, Low SES, M=20 | By Location | Exposed population had higher serum creatinine, p<0.05 |
| 2 | Alexander et al | 2014 | Illinois | Ecological | 20 | Natural gas plant, not specified | kidney cancer | Urban High SES total cancer deaths 5611(kidney =136). Reference Urban, High SES Total cancer deaths 27,170 (kidney=618) | By location | The AMR of cancer of kidney and pelvis in the exposed compared to less exposed counties was decreased [RR=0.97 (0.80,1.17) p>0.05]. Age, sex and race adjusted incidence rate of kidney cancer was also lower in exposed county [RR=0.91(0.81,1.01) P>0.05] |
| 3 | Benedetti M et al | 2017 | Italy | Ecological | 4 | PM2.5, cadmium | kidney disease (ICD-9-CM) | 4 exposed districts. Urban High SES. M=438, F=374. Reference: Urban M=879 F=671 | Model of air dispersion (SWIFT, SURPRO, SPRAY) | Excess of hospitalisation among men 20-59yr in high exposed area SHR=1.28(1.03-1.57) No excess in persons 60years + and in females |
| 4 | Bulat et al | 2011 | Serbia | Ecological | 3 | SO2, NH3, NO, CO,O3,PM, benzene, toluene, methyl mercaptane | kidney cancer | Urban, new cases n=3.8. Reference city, Urban n-177.8 | Air monitoring data. High /Low approach | Incidence of kidney cancer lower in exposed pop. SIR 3.7 compared to reference town 6.4. P<0.01 for males. Females SIR =3.1 vs. 3.5 P=<0.05 |
| 5 | Egwurugwu | 2013 | Nigeria | Cross-sectional | 0 | Gas flare, Not specified | serum creatinine | Rural, Low SES Total 475, Reference town: Rural, Low SES, total 315, M;F ratio 1:2 | By location | Exposed population had higher serum creatinine, % difference=13% |
| 6 | Egwurugwu et al | 2013 | Nigeria | Cross-sectional | 0 | Gas flare, Not specified | blood pressure | Rural, Low SES, Total 475, Reference town Rural, Low SES, total 315, M; F ratio 1:2 | By location | Exposed population had higher SBP (3.85% difference), DBP (7.55%) and MAP (5.82%) |
| 7 | Ejimofor et al | 2016 | Nigeria | Cross-sectional | 0 | Gas flare, Not specified | Hypertension  (blood pressure or use of anti-hypertensive | rural Low SES M=417, F=619, Reference town Rural, Low SES, M=454, F=535 | By location | Exposed population had higher odds of hypertension OR 4.85(1.84,12.82) |
| 8 | Garcia-Perez | 2016 | Spain | case-control | 0 | VOC, PAC, PM | childhood renal tumors | Urban. 213 (M=101, F=112) cases, 1278 (M=606 F=672) matched controls. | Industrial Pollution database | Increased risk for cancer observed for children living close (<2.5km) to organic chemical industry OR=2.22(1.15,4.26), PAC OR=2.16(1.16,4.03), VOCs OR=1.90(1.08,3.35), PMs OR=2.04(1.16,3.59) ; no significantly increased risk for refineries and coke ovens ,OR=1.73(0.35,8.53) |
| 9 | Haerey et al | 1980 | California | Ecological | 7 | petrochemical, not specified | kidney cancer | Urban High SES n=141,360. Reference: Urban, High SES , n=813,510 | by location | For men, the mean annual ASIR for kidney cancer was higher in exposed area compared to control areas (10.4 and 7.0 respectively per 100,000 SFBA population. p<0.05); lower incidence rate in females living in exposed areas compared to control area (2.5 and 3.0 respectively) |
| 10 | Hurtig et al | 2002 | Ecuador | Ecological | 13 | oil exploration, not specified | kidney cancer | rural Low SES n=118,264 , 55% M. Reference town: Rural, Low SES, n=155,710, 52.4% M | by location | An increased risk for kidney cancer for men in exposed counties compared to non-exposed RR=9.2(1.03, 82.20) |
| 11 | Idavain J et al | 2020 | Estonia | Ecological | 23 | Oil shale, Not specified | kidney cancer | Urban Mid-SES M=31,002, F=36,228. Reference Urban, Mid SES M=49,189, F=58,263 | By Location | The ASIR for kidney cancer in males almost unchanged and lower than reference town on average. In females, incidence 5.7% per year compared to 6.1% per year in non-oil shale areas. P<0.05 |
| 12 | Kaldor et al | 1984 | California | Ecological | 8 | Petrochemical emissions, SO2, HC, oxides of nitrogen | kidney cancer | Urban High SES M=787,185, F=809,588. Reference Urban, High SES M=1,443,751, F=1,483,446 | Model of air dispersion (BAAQMD) | Among exposed males, Age-adjusted incidence rate for medium and high exposed areas was 12.7 and 10.1, compared to State (8.8) and Country estimates(8.6) P<0.05. Not observed in females |
| 13 | Karadzinska-Bislimovska et al | 2010 | Marcedonia | Cross-sectional | 0 | Petroleum refinery and traffic emissions. Not specified | blood pressure | Rural Agricultural workers, Low SES, n=60, M/F ratio=1.4. Reference population Rural n=59 M/F ratio=1.1 | By location | Slightly higher mean DBP in the exposed population but not significant P=0.057, Mean SBP was not significantly higher (P=0.12) |
| 14 | Maduka et al | 2017 | Nigeria | Cross-sectional | 0 | Gas flare, Not specified | blood pressure | rural Low SES M=178, F=297, Reference town :Rural, Low SES, M=148, F=289 | By location | Exposed population had higher blood pressure OR 1.75 (1.11-2.04) |
| 15 | Ngwu et al | 2019 | Nigeria | Cross-sectional | 0 | Gas flare, Not specified | blood pressure, blood glucose | Rural, Low SES, M=183, F=240. Reference town: Urban, Middle SES, M=157, F=280 | By location | Exposed population had higher blood pressure, and blood sugar P=0.01 |
| 16 | Odo et al | 2019 | Nigeria | Cross-sectional | 0 | Gas flare, Not specified | serum creatinine, cystatin C | rural Low SES total 200, Reference town: Rural, Low SES, total 200 | By location | Exposed population had higher serum creatinine and Cystatin C (P<0.05) |
| 17 | Orru et al | 2018 | Estonia | Ecological | 0 | benzene, phenol, PM | hypertension, diabetes | Urban, Low-Mid SES, F=508 Reference: Urban, Mid SES, F=1535. Overall n=2127 | Air monitoring data | People living in quartile with highest level of exposure did not have higher odds of diabetes 1.01(0.85,1.21) or hypertension 1.10(0.98, 1.24) |
| 18 | Ovuakporaye et al | 2019 | Nigeria | Cross-sectional | 0 | Gas flare, Not specified | blood pressure | Rural, Low SES M=564, F=444. No Reference, all exposed communities | By location | higher DBP and SBP in some communities with exposure duration 1-5, 6-10years, but not >10years |
| 19 | Pirastu R et al | 2013 | Italy | Ecological | 12 | Industrial emissions, Not specified | Renal diseases, Kidney cancer | 4 exposed districts. Urban High SES M=48,759F=51,138. N= Reference M=108,272 F=113,187 | By location | In females, excess mortality risk for kidney cancer in 1 out of 5 districts studied (HR=3.68 CI;1.62,8.45); for men, excess risk in one district [HR=3.69(1.69,8.41)].  Excess hospitalisation risk from kidney cancer among females in 1 out of 5 districts studied, [HR 2.10(1.21, 3.66)]. For men excess risk in one district [HR 1.47(1.08, 1.24)]. |
| 20 | Ribeiro et al | 2016 | Brazil | Cross-sectional | 0 | Industrial sources (PAH, organochloride pesticides, PCB, dioxins and furans | hypertension | 4 exposed urban Mid -High SES n=4705. Reference: Urban, Mid-High SES n=1320 | By location | Living in one of the two exposed communities was an independent risk factor for hypertension; OR 1.3(1.0,1.6) and OR 1.4(1.1,1.8) respectively |
| 21 | Salerno et al | 2012 | Italy | Ecological | 7 | Petrochemical, not specified | kidney cancer | Urban, High SES n=6980. Reference: Urban, High SES | By location | For men, the risk for kidney cancer was lower in exposed area compared to control areas, SIR= 0.65(0.0, 1.56); in women, the risk was higher but not statistically significant, SIR=1.5(0.12, 2.88). Residents of exposed areas had an increased risk for kidney cancer-RR: 4.28(1.16-21.1) |
| 22 | Tsai et al | 2004 | Louisiana | Ecological | 30 | Petrochemical, not specified | diabetes, heart disease, kidney & bladder cancer | Urban, High SES. Reference Urban, High SES | By location | The AMR for kidney cancer among non-whites was not significantly different compared to reference pop (State and USA), but significantly lower or higher for white males depending on decade of study (80s or 90s). AMR for diabetes was significantly higher compared to reference population |
| 23 | Yang et al | 1997 | Taiwan | Ecologic | 9 | Petrochemical, not specified | kidney cancer | Urban, High SES M=977, F=853. Reference Urban, High SES M=870, F=758 | by location | AMR for kidney cancer among males was lower in exposed compared to reference town (RR=0.73 (0.43,1.22). In females, mortality rate was higher among exposed but not significant [1.17 and 0.73, RR=1.60(0.59,4.36)] |
| 24 | Yuan et al | 2020 | Taiwan | Cross-sectional | 4 | Petrochemical - arsenic, PAH | GFR, serum creatinine, CKD | Urban, High SES n=669. Reference: Urban, High SES n=1400 | By location and urinary content of metals | Living in HE areas associated with lower eGFR (β: 3.18; 95% CI: 2.08, 4.28) and higher odds of having CKD (OR 1.68; 95% CI: 1.32, 2.01)  eGFR was inversely associated with urinary concentrations of 1-OHP and arsenic. Higher CKD prevalence was associated with higher urinary concentration of arsenic (OR 1.14; 95% CI: 1.04, 1.26). |
| 25 | Yuan et al | 2021 | Taiwan | Cross-sectional | 0 | Petrochemical metals-nickel, chromium and vanadium | GFR, serum creatinine, CKD | Rural, Low SES, M=86 F=104, Reference town: Rural, Low SES, M=536 F=648 | By location and urinary content of metals | eGFR level of the HE group was lower than that of the LE group (β: -7.039; 95% CI: 9.000, 5.077; p < 0.05). The odds of CKD in the HE group were higher (β: 2.983; 95% CI: 1.946, 4.571; p < 0.05).  Urinary increase in nickel and chromium was associated with an increased prevalence of CKD and reduced eGFR |

M=Male, F=Female, LE=low exposure, HE=high exposure, SES: socioeconomic status, eGFR: estimated glomerular filtration rate, OR=odds ratio, HR= hazards ratio, SIR=standardised, AMR=adjusted mortality rate, RR=relative risk, incidence ratio.
