# Supplementary material for: Air Pollution and Chronic Kidney Disease Risk in Oil and Gas- Situated Communities: A Systematic Review and Meta-Analysis
Source: Int J Public Health. 2022 Apr 11;67:1604522. doi: 10.3389/ijph.2022.1604522 (PMC9035494; doi:10.3389/ijph.2022.1604522)
Supplement: Supplementary file 2 [file DataSheet1.pdf]

Ogo - A Systematic Review of the Epidemiological Literature Assessing Kidney-related Health Outcomes among Residents of Oil and Gas Situated Communities.

Medline and CINAHL, CAB Abstracts and Greenfile databases - combined search [EBSCO search platform]

- Go to my Libguide https://libguides.napier.ac.uk/shsc
- Select **Medline** from the left hand menu. Log in if asked with your student ID and password.
- Go to **Choose Databases** above the search boxes.
- Tick **all** these databases : **Medline** (the main medical database), **CINAHL** (the main nursing database), **CAB Abstracts** (environment) and **Greenfile** (environmental database)
- Type in the search as given below, including the punctuation.

Search 1 CKD + pollution + Africa

renal insufficiency chronic OR chronic kidney disease OR kidney failure OR end stage renal disease

AND

Particles OR "particulate matter" OR "sulfur dioxide" OR "sulphur dioxide" OR "nitrogen oxide" OR "nitrogen dioxide" OR "carbon monoxide" OR "ozone" OR air OR gas OR oil or petroleum

AND

*pollut**

AND

africa OR sub saharan africa OR global OR low income OR middle income OR developing countries

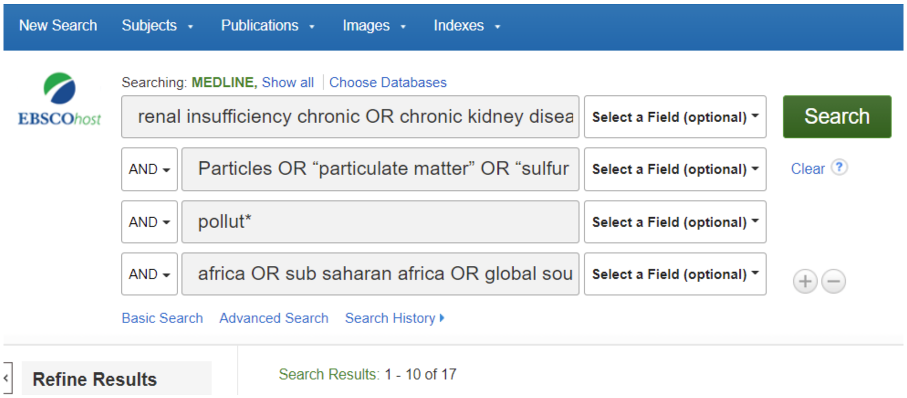

This gives 17 results. Scan the titles and abstracts.

Search 2

As search 1 but change the last line (geographic search terms) to the word “global” only

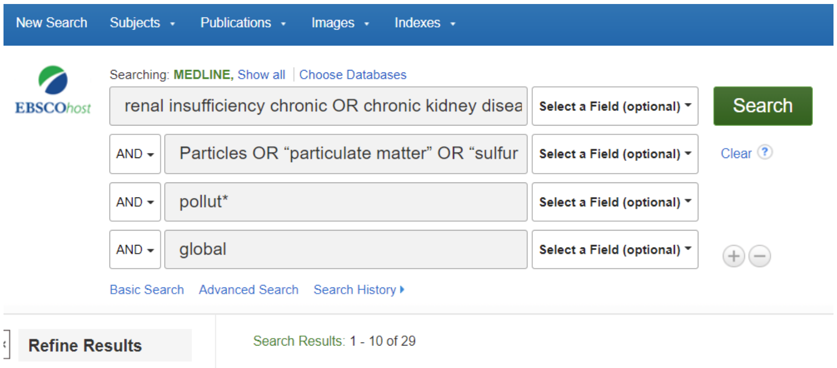

This gives 29 results. Scan the titles and abstracts.

Search 3 CKD + pollutants

Remove the last line from Search 1 (geographic search terms)

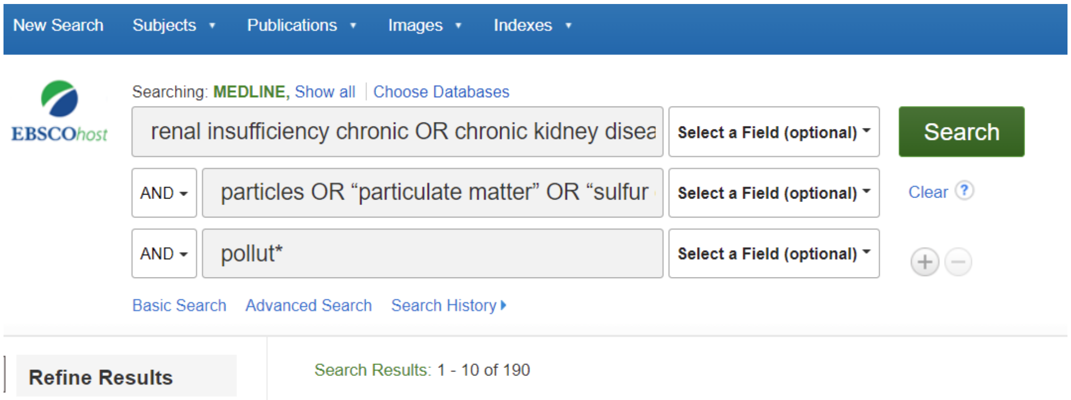

This gives 190 results. Scan the titles and abstracts

Search 4   NCD + pollutants

Remove CKD search terms

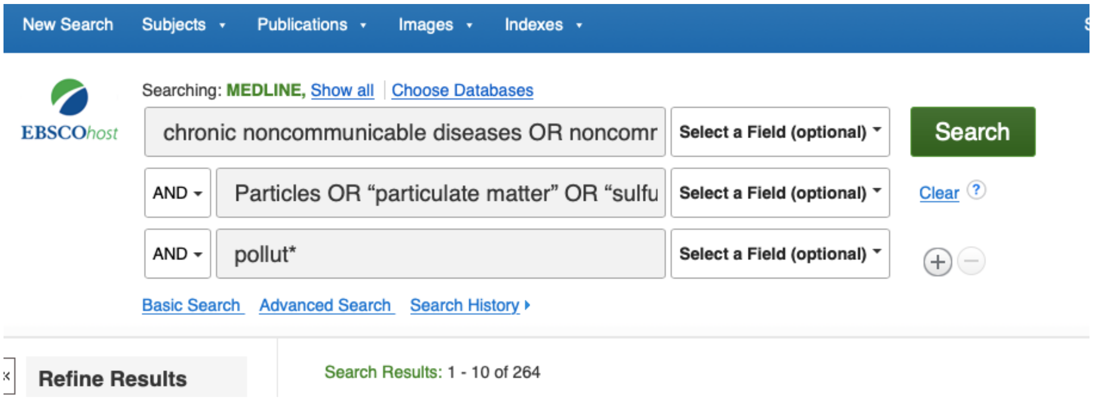

This gives 264 results. Scan the titles and abstracts
